# Engaging Councillors to Address Structural and Social Drivers of HIV Infections in Blantyre City: A Formative Study

**DOI:** 10.34172/ijhpm.8550

**Published:** 2025-06-09

**Authors:** Edna N. Bosire, Victor Mwapasa, Emily Mendenhall, Sara Allinder, Deborah Hoege, Pilira Chirambo, Emmanuel Kanjunjunju, Gift Kawalazira, Yohane Kamwigira, Tione Chilambe, Charles B. Holmes

**Affiliations:** ^1^Brain and Mind Institute, Aga Khan University, Nairobi, Kenya.; ^2^Center for Innovation in Global Health, Georgetown University, Washington, DC, USA.; ^3^Department of Public Health, Kamuzu University of Health Sciences, Blantyre, Malawi.; ^4^Edmund A. Walsh School of Foreign Service, Georgetown University, Washington, DC, USA.; ^5^Blantyre City Council, Blantyre, Malawi.; ^6^Blantyre District Council, Blantyre, Malawi.; ^7^National AIDS Commission, Lilongwe, Malawi.

**Keywords:** Structural Drivers, HIV and AIDS, Councillors, Blantyre City, Malawi

## Abstract

**Background:**

Blantyre city is among the jurisdictions in Malawi with the highest rates of people living with HIV and new HIV infections, driven by numerous structural factors. The Malawi National AIDS Commission hypothesized that local elected officials may be uniquely positioned to understand and address structural drivers of HIV infection in their communities. However, these leaders have been disengaged in HIV prevention efforts over time. This formative study aimed to explore city councillors’ understanding of the HIV landscape in Blantyre, including structural drivers of HIV, and to identify opportunities for engaging elected city councillors to address these drivers.

**Methods:**

Between November-December 2021, we conducted a descriptive qualitative study in Blantyre city, involving 59 purposively sampled participants: 23 city councillors, 14 technical experts, 7 implementing partners, and 15 community leaders. Data were collected through in-depth interviews and analysed thematically using MAXQDA software.

**Results:**

HIV technical experts and implementing partners were generally knowledgeable about the current HIV epidemic in Blantyre while most councillors and community leaders were not. Nearly all participants referenced structural drivers of HIV transmission in the city, including migration between districts, poverty, substance abuse, and transactional sex. Councillors noted their successes in mobilizing people and identifying resources for projects. However, they reported limited knowledge and training in HIV, no involvement in related programmes in their wards, and had minimal access to HIV data. They suggested access to trainings and data would equip them to better engage with HIV programs.

**Conclusion:**

Elected leaders in Blantyre have limited access to HIV data and training. However, they demonstrate well-established relationships with ward residents and possess motivation and interest in enhancing their knowledge and capacity to address structural and other drivers of HIV infection—key factors for designing interventions for local leaders.

## Background

**Key Messages**

**Implications for policy makers**

- Despite increasing efforts to ensure national leadership in shaping HIV programs, little attention is given to the roles of local leaders.
- Local leaders, such as elected councillors, are generally disengaged in HIV prevention efforts, often due to the prioritization of technical expertise in HIV programs.
- If trained with relevant skills, local leaders could offer a comparative advantage in HIV prevention, utilizing their access to communities and policy-making bodies. They could focus on addressing HIV structural drivers, by sensitizing communities about the epidemic and risk/stigma reduction, mobilizing resources, and passing and enforcing by-laws.

**Implications for the public**

We interviewed elected ward councillors, HIV technical experts, implementing partners, and community leaders to understand their knowledge of the HIV epidemic in Blantyre and thoughts on how to engage local leaders in prevention efforts. Our findings show that structural drivers- such as poverty, stigma and discrimination, gender-based violence, child marriages, health system challenges eg, distance to health facility contribute to HIV infections in Blantyre and can limit access to, and uptake of, services. Capacitating elected ward councillors through trainings and providing them with access to data, may be one way of identifying and addressing such drivers in Blantyre and other similar contexts in Africa.

Malawi has a substantial HIV epidemic, even though HIV prevalence has fallen and HIV treatment has broadened.^1^ Though estimated rates of new HIV infections in Malawi are declining, HIV risk remains high—especially in the south-west zone and Blantyre city.^1-3^ Structural drivers—the social, economic, policy, and organizational factors that increase the risk of HIV infection and inhibit access to, and uptake of, services. Studies in southern Africa, including Malawi, have identified structural risk factors to include challenges such as gender inequality,^4-6^ stigma and discrimination,^7^ child marriage, intimate partner violence,^4,8^ limited general awareness of HIV and AIDS and associated prevention methods.^9^ Poverty and low formal employment remains a driving structural risk for HIV, which is amplified among some of the most vulnerable residents of Blantyre, such as sex workers and truck drivers, who frequent drinking dens. Many healthcare workers identify condomless sex without pre-exposure prophylaxis (PrEP) and transactional sex, especially amongst adolescent girls and young women, to be a persistent challenge.^10^

 The Malawi Revised National HIV Prevention Strategy (2018-2020) outlined a multi-sectoral approach to HIV prevention in the country. This approach involved several steps, which included mostly biomedical interventions^11^ such as treatment as prevention, PrEP, condom, and lubricant programming—as well as behavioural change communication. Despite these efforts, it’s increasingly clear that such biomedical strategies have failed the residents of Blantyre and it is necessary to engage with local political leaders to take a broader frame for thinking about possible strategies to intervene in structural risk environments where high levels of HIV infection occur.

 Multi-sectoral teams for HIV prevention and management that are a well-coordinated systems at national, district and community levels can have an extraordinary impact. Siloed funding and reporting channels, however, often circumvent local government structures such as district-level leadership.^12^ This makes it difficult for ensuring that national level entities closely engage with community-based organizations, non-governmental organizations (NGOs), civil societies, and community health action groups. In this article, we attempted to design an experiment that may overcome these siloed cogs in order to pull together local officials learning about and in designing HIV prevention programs that transversed sectors and needs in the Blantyre city.

 As an effort to pilot possible solutions and workarounds for such siloed funding and to reinforce district-level coordination, the government of Malawi worked with civil society and other partners, supported by the Center for Innovation in Global Health at Georgetown University, to establish the Blantyre Prevention Strategy (BPS) in May 2020.^13^

 The Malawi National AIDS Commission proposed elected ward councillors would be uniquely positioned to support this last pillar in the prevention cascade, through identifying and addressing structural drivers of HIV within Blantyre city with the ultimate goal of encouraging sustained use of HIV prevention services. Historically, elected councillors in Blantyre had not been optimally utilized and were largely excluded from HIV prevention-focused governance structures and programme implementation.

 This formative study, therefore, was designed to investigate a range of stakeholders’ understanding of the HIV epidemic landscape in Blantyre, including the structural drivers of HIV, and to identify strategic opportunities to engage city councillors in addressing those drivers in Blantyre. In doing so, we aimed to strengthen district-level health system capabilities along the HIV prevention cascade by using data to target services targeting the highest risk groups. Our theory, that local officials know their constituent needs better than anyone, was placed at the center of this project and utilized to engage and design structural interventions based on community-led design and participation.

## Methods

###  Study Design and Setting

 This was a descriptive qualitative study conducted in Blantyre city, from November to December 2021. Blantyre is located in the southern part of Malawi and is the country’s commercial capital. Blantyre has the second largest population in Malawi after Lilongwe, with an estimated population of about one million people and a population density of 3328 persons per square kilometre.^14^ More than 70% of population in Blantyre live in urban centres, and 90% of its population is less than 49 years. The main government hospital that serves residents of Blantyre (Queen Elizabeth Central Hospital) provides free secondary and tertiary care to the population of Blantyre, including general medical services, HIV testing (provider-initiated testing and counselling) and antiretroviral therapy (ART). There are some smaller private (including private-not-for-profit) hospitals accessed by a small sub-set of the population who can afford the fees, but many people living in Blantyre access in patient care at Queen Elizabeth Central Hospital. Blantyre city has 23 wards, each with an elected councillor. Councillors are elected by city residents while the mayor is elected from among the councillors.

 Over the past two decades, Blantyre city has experienced a rapid population increase, as a result of rural-urban migration.^15^ This is in part because Blantyre forms the hub of industrial and commercial activities for Malawi and nearly all industries in the country.^16^ In addition, Blantyre is home to many tertiary institutions, including universities and technical colleges. As such, many young people from neighbouring districts relocate to Blantyre in search for employment or higher education.

###  Working With Elected Leaders in HIV Prevention Efforts

 Governance at the local level in Malawi takes the form of councils headed by a mayor or district commissioner, which include ward councillors, members of parliament, chiefs and appointed members representing special interest groups from within the local government area.^17^ Members of councils are elected for a five-year term to represent communities in the Local Authority to ensure that councils have structured mechanisms of accountability to communities by providing services equitably, effectively and sustainably within the means of the council.^17^ Some of the functions of the council include making decisions on governance and development for local government areas, promoting infrastructural and economic development, mobilising resources within the Local Government Area, and making by-laws.^17^ Thus, a councillor is elected to represent people in his or her ward in the council and is largely concerned with matters relating to services provided by the council to the people.

 Working with elected leaders presents a unique opportunity as they hold a position of influence but are also closely linked with their respective communities. For example, an elected leader can mobilize resources for their community through their membership on multisectoral governmental bodies, or by seeking the support of generous community members and organizations to sponsor programmes – such as youth or women empowerment programmes.^18,19^ Elected councillors are well-positioned to work with other local leaders given that they live within their communities, are well-respected in their communities, are involved in implementing community-based programmes, and play crucial roles in mobilizing people and resources.^20^ Elected councillors can also identify context-based and structural issues affecting their communities, and identify opportunities for innovative and visionary partnerships from within the community or leverage their involvement in making by-laws to address these structural issues.^17^

###  Study Population

 We interviewed diverse stakeholders from November to December 2021, including HIV technical experts, implementing partners, community leaders, and elected councillors to explore their understanding of the HIV epidemic landscape in Blantyre and the structural drivers of HIV infection. We purposively sampled 59 people to participate in in-depth interviews as follows: 23 elected city councillors who were recruited through leadership at the Blantyre city Council; 14 HIV technical experts (representatives from the District HIV and AIDS coordinating committee [DACC] and City AIDS coordinating committee [CACC]). We recruited these experts by liaising with relevant offices at Blantyre District and City respectively. We also recruited 7 participants from HIV implementing partners working in Blantyre – including Elizabeth Glaser Paediatric AIDS Foundation, Pakachere Institute of Health and Development Communication, Umunthu Foundation, and the Johns Hopkins Program for International Education in Gynecology and Obstetrics. Data in BPS’s integrated data pipeline and prevention adaptive learning management system (PALMS) was used to identify partners working in the district who were recruited for the study.

 Lastly, we identified four wards within Blantyre city characterized by high risk of HIV transmission from the BPS database. We liaised with councillors from these wards who helped us identify and recruit 15 community leaders. We purposively sampled 3-4 community leaders from each ward, who were stratified to include youth, men and women leaders.

###  Data Collection

####  Interviews

 Data were collected by two local trained research assistants and the principal investigator (ENB) for this study. First, we conducted a pilot study with three councillors. An interview guide with both closed and open-ended questions was used. Interviews began with a short survey about participants’ socio-demographic characteristics such as age, education status, and current roles. Thereafter, participants took part in an in-depth interview largely focused on health-related issues experienced in their communities including HIV and AIDS. This pilot study enabled us to identify and address gaps in our data collection tools and to develop questions that were culturally sensitive and appropriate. Interview guides were also translated from English to Chichewa—the primary spoken local language, for participants who preferred to use Chichewa.

 Thereafter, we started interviewing the councillors. Interviews with councillors were conducted in two private rooms at the Blantyre city Council. Most interviews were conducted in Chichewa. All interviews were conducted face-to-face and took an average of 60 minutes to complete. Next, we interviewed community leaders in a central public primary school or a local NGO in their wards. Afterwards, interviews with HIV technical experts were conducted. These experts included medical experts, administrators, finance representatives, and community representatives including religious leaders, and representatives of people with disabilities who were part of the DACC or CACC. All interviews were conducted face-to-face in the participants’ respective offices. Most interviews were conducted in Chichewa language and lasted for an average of 45 minutes. Lastly, we interviewed the various implementing partners working on HIV and AIDS programmes in Blantyre. These interviews were mostly conducted in English and online using Zoom meetings platforms. Interviews took an average of one hour to complete.

####  Observations

 We also conducted observations in the four wards where we interviewed community leaders. We did not have a structured observation guide but assessed factors such as the context where people lived, socio-economic activities, and proximity to basic amenities including schools, hospitals, and police stations. The research assistants and principal investigator documented field notes based on overall interviews and observations at the end of every day. These field notes helped triangulate the data from interviews and provided a detailed context of livelihoods in these wards.

####  Positionality and Reflexivity

 Our research team included both Malawian and non-Malawian researchers and stakeholders such as officials at the Blantyre District and City health offices, and we acknowledge that our positionality may have shaped participants’ responses and our interpretations. Local research assistants conducted most interviews in the local language, and regular reflexive discussions were held to reduce interpretive bias. Also, working with elected councillors was initially perceived to be difficult by researchers. This was in part because, being political leaders, there is a general perception of them being untrustworthy and unreliable. In addition, there is a perception of financial expectations when engaging these leaders. However, we clearly explained the scope of the research project at the beginning, highlighting that the project was not based on monetary incentives. We sensitized them on how they could leverage on their roles as elected leaders to support their communities as far as HIV prevention was concerned. We worked towards building a good rapport early on – something that they appreciated, which enhanced their support of our research without asking for money. Our collaborative approach that involved working with the locals helped ensure that there was no misinterpretation of the contextual issues reported. We learnt that if properly engaged and sensitized, elected leaders can be supportive of public health projects.

###  Data Management and Analysis 

 All interviews were audio-recorded. Audio files were transcribed verbatim, while translating directly from Chichewa to English. Initially, field notes and early interviews with participants provided a starting point for the development and definitions of an initial codebook. The principal investigator and one research assistant developed the codebook, which was uploaded in MAXQDA 2020 (version 20.4.2) software where overall coding and thematic analysis was conducted. Codes were developed both inductively (from the data itself) and deductively (based on research questions).^21^ The codebook was reviewed by one other researcher involved in the study (EM). The team then discussed the codebook and code definitions, then reviewed and revised codes based on mutual agreement. PC and ENB worked individually in reading and coding all the transcripts. Once complete, they met to discuss and resolve any coding discrepancies. Key identified themes were shared and discussed with all other researchers involved in this study. We then produced and reviewed the final theme classification under the following four broad themes with various sub-themes: (*i*) Knowledge of HIV epidemic landscape in Blantyre; (*ii*) Structural factors that influence HIV infection in Blantyre; (*iii*) Opportunities and gaps of engaging elected councillors in HIV response strategies; (*iv*) Suggested areas of support for the councillors. We reviewed and selected verbatim quotes from participants to provide examples and to illustrate the themes discussed in the results section.

## Results

 A summary of socio-demographic characteristics of respondents is provided in Table. Councillor ages ranged from 25 to 59 years, and nearly all (20/23) lived within their respective wards. HIV technical experts worked in various professions such as healthcare (nurses, clinicians, public health) and community development workers such as representatives of disability groups, religious leaders, and NGO project officers, among others.

**Table:** Participant’s Socio-demographic Characteristics

**Sample socio-demographic characteristics**	**Councillors (n = 23)**	**Community Leaders (n = 15)**	**DACC, CACC (n = 14)**	**Implementing Partners (n = 7)**
25-35	13% (3)	20% (3)	21% (3)	0% (0)
36-45	26% (6)	20% (3)	43% (6)	71% (5)
46-55	39% (9)	33% (5)	14% (2)	29% (2)
56+	22% (5)	27% (4)	22% (3)	0% (0)
Gender				
Men	87% (20)	73% (11)	57% (8)	71% (5)
Women	13% (3)	27% (4)	43% (6)	29% (2)
Education				
No school/primary school	0% (0)	60% (9)	0% (0)	0% (0)
Secondary school	65% (15)	40% (6)	22% (3)	0% (0)
Technical school/college/university	35% (8)	0% (0)	78% (11)	100% (7)
Work experience (y)				
1-5	13% (3)	13% (2)	14% (2)	0% (0)
5-15	30% (7)	53% (8)	22% (3)	43% (3)
Over 15	57% (13)	33% (5)	64% (9)	57% (4)

Abbreviations: DACC, District HIV and AIDS coordinating committee; CACC, City AIDS coordinating committee.

###  Theme 1: Knowledge of HIV Epidemic Landscape in Blantyre

 Although councillors and community leaders demonstrated basic understanding of HIV and AIDS, they lacked a comprehensive familiarity with current data on the local epidemic. Councillor 8 explained, *“I can’t say that I have enough information on HIV and AIDS, all I know is that things are not good, and it needs to be addressed”* (30-year-old male). They also lacked a clear understanding of populations at risk in their wards or communities. All councillors reported that they were not aware of the prevalence of HIV in their wards.

 Poor HIV epidemic knowledge among councillors was attributed to limited HIV sensitization in the communities. Councillors provided examples such as limited posters or billboards with HIV and AIDS information on roads, markets, or other common spaces. Additionally, though community focus prioritizes disease areas such as high blood pressure, cancer, and diabetes, HIV and AIDS prevention has fallen from among these priority areas, despite persistent rates of new infection and high prevalence. In fact, one implementing partner working on HIV programmes said that provision of ART medication and PrEP had made people feel that HIV is no longer a threat. It was also evident that there was a lack of understanding and even negative sentiments towards usage of HIV prevention methods such as PrEP, especially amongst the councillors. For example, one councillor reported that; “*The PrEP that is currently being provided is what is promoting careless sex” *(40-year-old-male).Other participants echoed such sentiments saying that people were engaging in unprotected sex because they could use PrEP to protect themselves from HIV infections. One participant described how possible HIV infection was now becoming accepted/normalized at the community level saying: *“Some people at the community have literally normalized HIV infection. With ARVs they regard it as something that is normal and there was nothing wrong about having HIV” *(62-year-old mal, community leader).While community members and leaders demonstrated a clear understanding of the effectiveness of ART in treating HIV, they also displayed a lack of understanding of prevention methods, such as PrEP, and the urgency around which these interventions are needed.

 Other study participants, such as HIV technical experts and implementing partners, demonstrated greater familiarity with the existing HIV epidemic in Blantyre, including an understanding of epidemiological data and an appreciation for the critical role that prevention plays in the future of the epidemic. This level of familiarity could be attributed to their active participation in implementing prevention and care and treatment programs and routine access to data.

###  Theme 2: Structural Factors That Influence HIV Infection in Blantyre

 We sought to explore the structural drivers of HIV infection in Blantyre city. Our participants were able to identify a number structural drivers of HIV infection in Blantyre, consistent with other previous studies conducted in Malawi.^7,22,23^ We discuss some of these results below:

####  Poverty, Rural-to-Urban Migration and Ease of Mobility

 Participants noted that Blantyre city is home to major industries, such as manufacturing and tobacco industries, and tertiary institutions, which has influenced rapid growth and expansion of the city.^16^ Participants also cited poverty and a need for increased job opportunities or education as driving factors for why many people, especially youth, are moving from neighbouring towns and districts such as Mulanje, Zomba, or Chikwawa to Blantyre. However, it was also noted that due to the large influx of residents from other neighbouring districts, many youth are unable to secure jobs or education upon arrival in Blantyre – which leaves them unable to meet their basic needs. One participant described:

 “*Migration from other districts such as Mulanje to Blantyre is becoming common. Some people end up struggling if they can’t get a job; their children end up dropping from school and young people find themselves in vulnerable situations for transactional sex*” (51-year-old male, councillor).

 Participants revealed that in the context of unemployment and poverty, individuals engage in transactional sex as a means to meet basic needs such as food and shelter in Blantyre:

 “*Many of those who migrated to Blantyre and have not secured a job have turned to selling their bodies in order to afford the next meal*” (37-year-old female, councillor).

 “*When I ask the women to stop selling their bodies, they usually ask me, are you going to provide food for my children?*” (54-year-old female, community leader).

 We also learnt that there is a sizeable and mobile population that travels for commerce and stops in Blantyre in transit to other districts in Malawi and other countries. Rest houses have been established in the frequented stopover areas to cater for this population. One participant shared:* “These truck drivers find cheap accommodation in places like ward x. They stop by for a sleep over and end up luring women into sexual relationships because they always have disposable income from their daily allowances” *(50-year-old male, councillor).

 Moreover, some local transportation systems such as motorcycle riders—locally known as *“Kabaza*” were also reported to have expendable cash in Blantyre as described by participants; *“Kabaza riders usually operate 24 hours. These boys are hired by sex workers as a means of cheap transport to and from their houses. Sometimes, these drivers end up having sex with the sex workers” *(38-year-old male, councillor).These activities were compounded by motorcycle riders having expendable money from daily income, and availability of alcohol, lodges and individuals engaging in transactional sex.

####  Substance Abuse, and Unregulated Shebeens and Rest Houses

 Cheap and easy access to alcohol and other substances at the community level were reported as key drivers of behaviour that increase risk of HIV (eg, condomless sex without PrEP) in Blantyre city. Respondents suggested alcohol and substance use serve as coping methods for youth dealing with poverty, unemployment, and the inability to finish school. One participant said, *“It is common now to see young men into alcohol and substance abuse due to frustrations. Others are smoking chamba [weed] and other hard drugs” *(48-year-old male, community leader).Another participant said,* “HIV has greatly affected community x because many women and young girls disappear into the drinking dens where they drink and have sex to get money” *(38-year-old male, councillor).

 Closely linked to alcohol and substance abuse was the rampant mushrooming of *“shebeens” *– unregulated drinking dens and bottle stores. In two wards, we observed many shebeens where we could see youths playing board games, such as chase and pool table, inside while drinking alcohol. Councillors and community leaders explained about the increasing numbers of shebeens in Blantyre city, which they believe has led to increased alcohol and substance use. Community leaders also noted that in recent times, there has been an increase in establishment of unregulated residential shebeens – where backyards of residential spaces are being converted into drinking dens – something that we also observed during our walks in various wards. This was said to be common in places where local brew “*Kachasu” *or bitter opaque beer* “Ntonjane” *were produced and consumed. One participant said, *“…these residential shebeens are becoming rampant and have become a meeting place for cheap alcohol and sex amongst young boys and girls” *(55-year-old male, community leader).

 Moreover, participants reported that there were many cheap and easily accessible rest houses and lodges in most populated and low-income areas in Blantyre city, which also facilitated behaviour that increases risk of HIV (eg, condomless sex without PrEP). Similar to shebeens, participants narrated how rest houses and lodge businesses were thriving as owners made quick money through “short term” bookings especially by clients involved in transactional sex. A participant said; *“In community x there are lodges that are rented out by sex workers who migrate to this place for a month to 3 months, with an aim of having a readily available space for sex” *(50-year-old male, councillor).

 Observation: We accompanied a HIV implementing partner to the community – and observed how they engage with bottle owners. We observed and learnt about how rest houses operate during the day – with women from young girls to middle aged women sitting outside these rest houses or doing laundry within these spaces while waiting for potential clients for transactional sex. We informally chatted with the women, and they revealed to us that they do transactional sex.

 All together, we learnt that commercial-related mobility, poverty, easy access and availability of alcohol and substance abuse as well as cheap rest houses influenced transactional sex in Blantyre city. Councillors were concerned about how transactional sex had become rampant in their wards as demonstrated by a participant: *“Many young girls are getting involved into sex work to earn a living. This is also becoming common amongst college and university students*” (50-year-old male, councillor).

####  Stigma, Low Sensitization About HIV and AIDS in Blantyre

 Stigma was also discussed as another driving factor influencing HIV infections in Blantyre city. A participant said, *“People living with HIV are highly stigmatized”* (45-year-old, male, HIV technical expert). Many participants noted that stigma was the reason why many people avoided open discussions about HIV and AIDS, as explained by another participant, *“some families are unwilling to openly discuss about HIV with their children due to the stigma. This is a gap especially in homes with teenagers or youths who have started engaging in sex” *(38-year-old male, Councillor). Furthermore, community stigma against those known to be living with HIV and AIDS was said to be a factor that dissuaded many from accessing healthcare. Stigma was particularly said to be rampant towards men who have sex with men and individuals engaging in sex work – and this happened both at community and health facility levels. One participant explained that:* “Because homosexuality is still illegal in Malawi, this group of people are usually stigmatized. Many end up hiding and not seeking care or preventive products such as lubrication or PrEP” *(56-year-old male, HIV technical expert).

 Our findings also reveal that stigma was compounded by the previously mentioned low HIV and AIDS sensitization in communities, and individuals lacking information on what steps they can take to protect themselves from infection.

####  Gender Inequality 

 Gender inequality was also linked to HIV transmission in Blantyre. For example, gender-based violence, sexual assault, as well as some cultural practices where young girls are married off to older men were commonly reported by respondents. A participant said, *“…these are harmful cultural practices where girls are forced to drop out of school and getting married off to older men, which creates vulnerability for HIV infections”* (A female, HIV implementing partner). Importantly, gender differences were widely discussed in relation to cultural beliefs and practices where women were perceived to be powerless in matters around sex and decision-making; *“Mostly, men are key decision makers including on matters to do with sex. That’s how women end up having unprotected sex*” (A male, HIV implementing partner); or “*women are silenced in exchange of food and shelter*” (A female, HIV implementing partner). In addition, it was reported that unemployment and lack of education amongst women reinforced this gender power dynamic that led to sexual exploitation.

####  Health Systems Challenges 

 Participants also cited concerns with gaps in HIV services for people living with HIV that may impact viral suppression rates. First, drug stockouts or inconsistent dispensation of ART was reported to be a challenge. For example, one participant mentioned that *“Although hospital x was built and set aside for ART dispensation, it has not been operational and there are no ARTs distributed. People, including youths, are forced to get their ARTs from other health centre[s]*” (63-year-old female, community leader). Respondents also expressed concern with the eventual phase-out of external partner-supported programs and how this might further impact consistent access to ART.

 Another barrier to HIV service access that was identified is the distance to healthcare facilities. A participant said,“*In my ward, we do not have a health centre or a mobile clinic. People must walk for at least 10 km to and from their homes to get treated” *(35-year-old female).Another participant said: *“The nearest health facility is in community x which is 10 km away from our organization. As a result, some people default from their ART medication because of distance to hospital. Others are unaware of their status, even though they continued to engage in risky sexual practices”* (A female, HIV implementing partner). As demonstrated by the councillor and implementing partner, frequent defaulting of ART could lead to a risk of lower rates of viral load suppression.

###  Theme 3: Opportunities and Gaps of Engaging Elected Councillors in HIV Response Strategies

####  Councillors’ Current Roles

 First, we sought to understand councillors’ current roles and responsibilities in their wards. Councillors referred to themselves as “voluntary leaders” – meaning they were selfless in serving their communities. One councillor explained, “*I am involved in listening to people and taking their concerns to the council while at the same time, channelling the developmental ideas from the council to the people that I serve” *(55-year-old male, Councillor). The fundamental role named by all councillors was the same: to represent their community’s interests at the council. In addition, councillors added that they lived within their wards and were critical in identifying community challenges, advocating for and representing their communities at the Blantyre city Council, exemplified by the following councillor’s description of role: *“As a councillor, in actual sense they say that you are a volunteer, but my work on a daily basis is to help any problem that arises in my ward, whether the roads have gone bad, or there is need to build a bridge, but also taking care of the sick” *(51-year-old male, Councillor).

 Another important element of their roles is initiating developmental projects at the community. Almost all councillors discussed the various developmental projects that they were working on, such as construction or rehabilitation of bridges, toilets and taps at schools, roads among others – activities that has gained their trust amongst the communities they served. For instance, a participant explained, *“I am a community leader as my job description says, I am also a community mobiliser, and my duty is to coordinate with government in terms of development” *(38-year-old male, Councillor).Many councillors noted that they were key in mobilizing financial and human resources. A participant said,* “If there is an NGO that comes through a councillor, it gets a lot of attention. People respect whatever the councillor says” *(56-year-old male, Councillor).During discussions, the issue of mistrust from community towards councillors (as politicians) came up. Many politicians are perceived as people who do not keep their words – in terms of commitment to the electorates. However, almost all councillors mentioned that their various development track records in the wards had earned them respect. In addition, they revealed that they had established a good working relationship with other existing leaders and existing local structures in identifying community challenges and solving these challenges together.

 “*If a message is taken to the community through us, it spreads faster because we already have ways of disseminating it, we work with other community structures such as the CDC (Community Development Committee). This makes mobilization of people so easy” *(44-year-old male, Councillor).

 We learnt that councillor’s roles involve supporting socio-economic, political and health aspects of the people they served. Thus, they worked closely with a myriad of community organisations or authorities to ensure the comprehensive well-being of the people in the communities.

####  Existing Opportunities for Engaging Councillors in HIV Prevention Efforts

 Councillors reported that they are always provided with platforms and expected to address community members such as during funerals, churches, and chiefs’ meetings. They also revealed that they are respected in their communities and their word was taken seriously by community residents. These platforms could serve as an untapped mechanism through which councillors can begin sharing updated and ward-specific information with their communities about the local HIV epidemic.

 Moreover, we learnt that some councillors were leading various sports activities in their wards such as football and netball games for boys and girls as exemplified by a participant who said, *“I have established two football teams for girls and boys with an aim of keeping them busy while also growing their talents” *(63-year-old male, Councillor). Other councillors reported that they were instrumental in organizing periodic events in their communities such as HIV and AIDS days, youth days among others. As such, these existing activities could be reinforced with relevant HIV prevention messaging to reach many young people in their communities.

####  Gaps in Involving Councillors in HIV and AIDS Programmes in Blantyre

 Councillors revealed that they were rarely involved in health-related programmes, including HIV and AIDS-focused projects. One councillor noted, *“We are hardly involved [….]. I know there are many organizations that are working at facility x, but as a leader, I have never been introduced to these organizations” *(55-year-old male, Councillor). For others, although they were made aware of the projects taking place in their wards, they were only engaged during project inception and not during the implementation phase. A participant said, *“In my ward, we have the clinics and HSAs [Health Surveillance Assistants] who work with communities. What is strange is how the HSAs do not want to involve me and the local leaders” *(50-year-old Male, Councillor).

 Some councillors argued that their exclusion from health-related projects was due to councillors being perceived as *“government’s spies” *who could report issues to do with misuse of resources. One councillor said: *“We are regarded as watch dogs or spies who are ready to report their wrong doings to the authorities” *(35-year-old female, Councillor). Consequently, non-involvement was perceived as the reason why some projects failed; *“*…*this exclusion makes the interventions to flop because it is me who knows how to talk to people in my ward” *(42-year-old male, Councillor).

###  Theme 4: Suggested Areas of Support for the Councillors

 When asked how they could be supported in HIV prevention efforts, councillors revealed that they desired to be more actively involved in HIV programs in Blantyre, including addressing the structural drivers of HIV infections. Other participants in this study, such as community leaders, shared similar thoughts and felt that councillors had a good understanding of their communities and would be instrumental, not only in HIV awareness creation, but also in designing solutions to problems that affected the people they served. Secondly, councillors reported a need to be provided with necessary information on HIV to enable them to engage their communities with accurate and real-time data, *“we need much support, especially information. There must be enough information sharing on HIV”* (44-year-old male, Councillor). In addition, councillors suggested that they should be trained and be provided with capacity building skills to facilitate proper engagement with their communities, *“I will say again that do not give us money but help us with the needed equipment. I would need capacity building trainings”* (48-year-old female, Councillor). These sentiments were also reflected in interviews with HIV technical experts and HIV implementing partners – who strongly recommended that councillors should be trained and provided with capacity building skills to enable them to engage with their communities in HIV sensitization, prevention, and other health messaging.

## Discussion

 This is the first study in Malawi to explore how local elected leaders can be engaged in HIV prevention efforts. We engaged with diverse HIV prevention stakeholders and triangulated these findings with observations in four wards within Blantyre city characterized by high risk of HIV transmission to cultivate rich insights into identifying structural drivers of HIV infection in their respective wards. City councillors are influential and creative in their communities, particularly when mobilizing resources to address structural drivers of HIV infection. However, we found they are underutilized when it comes to messaging about, providing insight for, and leveraging access to HIV prevention. This is a missed opportunity because councillors hold a familiarity with the unique structural drivers that impact each of their wards and can serve as critical advisors for government and implementers during HIV prevention program design phases. We argue that engaging local elected officials in Malawi could serve as an important opportunity to optimize ward-level HIV prevention efforts, galvanize community engagement around prevention, and utilize city council structures to tackle structural drivers of HIV.

 Structural drivers of HIV infection are critical in Blantyre city. We found important overlaps among structural drivers, including poverty, unemployment, food insecurity, and gender inequality, and social and behavioural factors, such as transactional sex and substance abuse, which together synergistically reinforce HIV risk in this context. These findings are consistent with previous studies conducted in Malawi^4,24^ and other African countries.^25,26^ Councillors provided important insights into this problem, revealing opportunities to leverage their positions on Blantyre city Council to actively respond to some of these structural drivers. For instance, supporting bylaws that prohibit early marriages and ensuring that vulnerable populations can get access to support and health messaging from government and other community-based organizations in their wards. In addition, councillors could partner with other community organizations and leverage resources from the multisectoral committees to boost socio-economic programmes.

 Many participants described a link between mobility and HIV infections in Blantyre. This was in part because of limited economic opportunities, especially in rural areas, lead young people to relocate to Blantyre city in search of employment or schools. Yet, some lose hope because work and school are hard to maintain, causing them to turn to alcohol and other drugs, which can at times influence risky behaviours that may heighten risk for HIV. Moreover, drinking dens and rest houses serve the many people who pass through Blantyre working in local transportation and commercial systems with disposable income. Substantial evidence suggests that HIV risk runs through these routes, escalating risk for transmitting the virus,^27,28^ often as a result of drinking and risky sex with multiple partners.^5,29^ These risk environments also amplify the likelihood of gender-based violence, especially when one or both sex partners have been drinking.^30-33^ Women in financially insecurity situations are particularly vulnerable in these cases (including when women are engaged in sex work). Councillors may serve these commercial areas by recognizing the risk environment and target public health programs and messaging (such as in drinking dens and rest houses where people spend their time) and ensuring law enforcement is available to keep women safe.

 Challenges accessing healthcare facilities and disruption of services were also concerns in Blantyre city, causing some residents to walk many kilometres to access government or public hospitals that provided HIV treatment free of charge. Recent studies from Malawi^34,35^ and elsewhere^36,37^ have attributed HIV “loss to follow-up” to distance to the hospital. This was particularly troubling when drug stockouts prevented routine HIV care.^38,39^ However, some care-seeking barriers were also cultural. Many people expressed fear of stigma within the community, which prohibited open dialogue around HIV prevention methods—including discussions around PrEP between parents and children and among partners, as well as perceived stigma in healthcare settings. This stigma was even more pronounced for female sex workers and men who have sex with men, which is consistent with findings from South Africa and in the USA.^40,41^ Addressing these biomedical or health system barriers in HIV prevention and treatment is paramount. Local leaders can be capacitated to support in creating community awareness about how and where people can access ARTs as well as PrEP for HIV treatment and prevention.

 Councillors live in their communities and understand the key challenges that affect their constituents. Tailoring HIV programs at the ward-level, for example, enhances the potential that structural drivers of HIV within programs may be identified based on community needs. Recognizing community ownership of HIV projects or programmes is essential for long-term sustainability.^42^ Such community-centred design would also enhance the possibilities of integrating multiple sectors into HIV programming, such as nutrition and farming. Given their prominent roles and access to city council activities, councillors can shape health policies that address HIV risks to communities, for HIV by making by-laws in the council.^17^ However, many participants urged for the need to train these local leaders so they are aware of local needs.

 These recommendations have shaped our next phase of activities. We plan to design and launch a city-led structural risk reduction working group comprised of councillors and other stakeholders that will enable real-time discussions and learnings about HIV prevention efforts in Blantyre. We plan to organize capacity building trainings for councillors on topics around understanding of HIV and AIDS, access to HIV prevention and treatment options such as condoms, PrEP and ART, as well as communication and resource mobilization strategies. These efforts will train councillors in developing innovative strategies that consider ward-specific structural drivers and needs to reduce HIV impacts in their wards. These skills may enhance councillors’ ability to collaborate with stakeholders across sectors to integrate their knowledge of HIV prevention efforts within ongoing activities at Blantyre city Council. Finally, we will introduce the PALMS to the councillors and train them to use it. PALMS is an integrated HIV prevention data pipeline and dashboard developed through BPS which brings together historically disconnected national and facility-level data. This ward-specific data will enhance councillors’ ability to make decisions around ward needs for HIV prevention efforts.

###  Study Limitations

 This study is not without limitations. We worked closely with local researchers and other stakeholders at the Blantyre city and District. However, we recognize that some participants may have felt constrained in voicing critiques of local leadership or HIV programming due to perceived power dynamics or future funding concerns. Also, we did not involve councillors who are primarily responsible for rural services within Blantyre District, although we hope to include them in our next phases of study. Moreover, all participants were recruited from Blantyre city, and their experiences may differ from other towns and cities such as Lilongwe and may not be fully generalizable. Nevertheless, the findings provide important insights in engaging local elected leaders in HIV response- an area that has not been explored before in Malawi.

## Conclusion

 This research reveals how a multiplicity of structural, social and behavioural factors drive HIV risks and infection in Blantyre city. For example, we found that transactional sex is common in Blantyre, and acts as a coping strategy in the context of poverty and gender inequality. As such women and girls are the most vulnerable. Multi-prong strategies are necessary to bolster existing HIV interventions, and we argue that elected officials, such as city councillors, may serve to be an untapped but strategic resource because they understand the needs and priorities of their ward residents. Engaging local leaders can promote community awareness of HIV risk reduction efforts and support equitable access to HIV prevention and treatment services. Councillors may also leverage bylaws and resources at the Blantyre city Council in order to mobilize city and private resources to bolster HIV prevention programming. Some of these bylaws and resources may address some of the most pressing community challenges, such as early child marriage, regulating drinking dens, and working with local implementing partners to deliver HIV prevention programming and condom distribution in the wards. Bringing together city council with clinicians and NGOs may further elevate these interventions and provide long-term solutions for structural risk environments that have long affected residents of Blantyre and beyond.

## Acknowledgements

 We are greatly indebted to the participants who took part in this study. We wish to acknowledge Rose Sakala for her efforts in data collection for this study.

## Ethical issues

 This study was conducted according to the guidelines laid down in the Declaration of Helsinki, and all procedures involving human subjects were approved by the College of Medicine Research Ethics Committee (COMREC; P.09/21/3407) in Malawi and the Institutional Review Board (IRB) at Georgetown University [IRB ID: STUDY00004570], USA. Written informed consent was obtained from the study participants after reading out the content of the information sheet and explaining the purpose of the study (for face-to-face interviews) while interviews conducted virtually, verbal consenting was captured in audio-recording.

## Conflicts of interest

 Authors declare that they have no conflicts of interest.

## Availability of data and material

 All data supporting this research is provided within the manuscript.

## Additional Information

 Our dear co-author Emmanuel Kanjunjunju, sadly died on July 20, 2023.
